# Neuronal extracellular vesicles biomarkers of Alzheimer's disease in a subset of a longitudinal cohort using plasma

**DOI:** 10.1002/alz70856_103966

**Published:** 2025-12-26

**Authors:** Darlene Antoine, Saswat Sahoo, Alexander Daskalopoulos, Tushar Dubey, Maja Mustapic, Apostolos Manolopoulos, Cynthia Munoz, Lon S. S. Schneider, Dimitrios Kapogiannis

**Affiliations:** ^1^ NIH(NIA), Baltimore, MD, USA; ^2^ Nih(NIA), Baltimore, MD, USA; ^3^ Lerner College of Medicine, Cleveland, OH, USA; ^4^ National Institute on Aging, Baltimore, MD, USA; ^5^ University of South California, Los Angeles, CA, USA; ^6^ Alzheimer's Disease Research Center, Keck School of Medicine, University of Southern California, Los Angeles, CA, USA

## Abstract

**Background:**

Amyloid beta peptide 40 and 42, tau and phosphorylation tau are the main pathological proteins in AD and have been targets for biomarker development. The ability to measure these molecules in biofluids could provide evidence of ongoing pathophysiology. Extracellular vesicles (EV) play a role in the spread of these pathological proteins, which may explain why associated biomarkers may be used to diagnose and track AD. Examining how distinct NEV biomarkers of AD such as distinct pTau species are associated with cognition as well as with each other, sex and age is essential to define their value as AD biomarkers.

**Method:**

This longitudinal study cohort includes 30 participants with AD from University of South California that were cognitively evaluated over time with RBANS. Plasma samples from 2 timepoints 6 months apart were used in this study. We immunocaptured neuronal extracellular vesicles targeting L1CAM, NLGN3, GAp43 and measured Ab40 and 42, and Total, p181, p217 and p231‐Tau. A linear mixed model was used to determine whether these EV biomarkers are associated with cognitive test scores, with and without including sex as a factor and age and education as covariates.

**Result:**

p231‐Tau was uniquely positively associated with RBANS (beta=0.035, *p*‐value = 0.0208). When age was used as a covariate in the model, total‐Tau was also positively associated (beta=0.1805p=0.0475). All pTau species were positively associated with each other, however a negative association was observed between Total Tau and p231‐Tau.

**Conclusion:**

Our findings show evidence of distinct pattern of associations between different pTau species and cognition, suggesting some unique properties of p231‐Tau in tracking disease severity in AD. This would suggest that p231‐Tau, one of the earliest phosphorylation to occur in the development of AD, may be best suited for monitoring disease activity in AD and for showing response to experimental treatments. Further basic research and application in large cohorts is needed to validate and elucidate the basis of this unique feature.

